# Localization and Diagnosis of Attention-Deficit/Hyperactivity Disorder

**DOI:** 10.3390/healthcare9040372

**Published:** 2021-03-27

**Authors:** Peng Wang, Xuejing Zhao, Jitao Zhong, Ying Zhou

**Affiliations:** 1 School of Mathematics and Statistics, Lanzhou University, Lanzhou 730000, China; wp17@lzu.edu.cn (P.W.); zhongjt17@lzu.edu.cn (J.Z.); zhouyingmath@163.com (Y.Z.); 2 School of Mathematics and Statistics, Huazhong University of Science and Technology, Wuhan 430074, China; 3 School of Information Science and Engineering, Lanzhou University, Lanzhou 730000, China

**Keywords:** attention-deficit/hyperactivity disorder, random forest, classification, disorder localization, threshold selection

## Abstract

In this paper, a random-forest-based method was proposed for the classification and localization of Attention-Deficit/Hyperactivity Disorder (ADHD), a common neurodevelopmental disorder among children. Experimental data were magnetic resonance imaging (MRI) from the public case-control dataset of 3D images for ADHD-200. Each MRI image was a 3D-tensor of 121×145×121 size. All 3D matrices (MRI) were segmented into the slices from each of three orthogonal directions. Each slice from the same position of the same direction in the training set was converted into a vector, and all these vectors were composed into a designed matrix to train the random forest classification algorithm; then, the well-trained RF classifier was exploited to give a prediction label in correspondence direction and position. Diagnosis and location results can be obtained upon the intersection of these three prediction matrices. The performance of our proposed method was illustrated on the dataset from New York University (NYU), Kennedy Krieger Institute (KKI) and full datasets; the results show that the proposed methods can archive more accuracy identification in discrimination of ADHD, and can be extended to the other practices of diagnosis. Moreover, another suspected region was found at the first time.

## 1. Introduction

Nowadays, increasing attention is being paid to brain sciences, especially concerning neurogenetics, memory function and cognitive function. Studies on medical imaging data have gained great popularity with the rapid development of various neuro-imaging technologies. Existing imaging technologies include magnetic resonance imaging (MRI), functional MRI (fMRI), electroencephalography (EEG), diffusion tensor imaging and positron emission tomography (PET) [1,2]. Large amounts of data from different imaging technologies urgently need to be analyzed. In recent years, there have been increasing numbers of studies on such medical data and their applications in clinical experiments. Koji and Yamada [3] gave a review of the trend of joint cooperation in multiple fields and the applications of machine learning.

Attention-Deficit/Hyperactivity Disorder (ADHD) is a common neurodevelopmental disorder regarding personal development among children that can last into adolescence and adulthood. Symptoms include difficulty in concentrating, and in controlling behavior and hyperactivity; these abnormal behaviors often interfere with normal individual development. Research and treatment of ADHD with machine learning methodology has been a hot issue for researchers in recent years [4]. In order to assist in diagnosing the disorder, two issues, classification and disorder-region detection, must be addressed. Lanka et al. [5] introduced the issue of data heterogeneity, which brings great challenges to related research and analysis. Duffy et al. [6] constructed a model on the clinical features and principal components of EEG data to complete a classification task. Moreover some methods were proposed to directly use single MRI data, such as the 3D-CNN method of [7], the tensor regression method of [8] and the social network method of [9]. Some methods of brain effective learning (BEL) were also applied to the research based on single MRI data. Mei and Tan [10] proposed updating the weights of amygdala and orbitofrontal cortex in emotional learning by a genetic algorithm based on BEL, and obtained higher classification accuracy. Some graph-network methods were applied to brain networks [11,12]. Intani et al. [13] extracted the image characteristics and clinical features of the brain, and the combination method was then used to construct a decision tree for the classification. Huang et al. [14] proposed a regression method to estimate the brain-lesion region. Goldsmith et al. [15] conducted a series of studies on the basis of image characteristics and clinical features, and further contributions to the construction of regression models. There are currently two main disorder-region-detection methods:

(1) Estimated coefficients were used to obtain the lesion region by using regression methods such as tensor regression and Tucker regression as proposed by Zhou et al. [8] and Li et al. [16].
(2) The region of interest is directly derived upon multi-classification, including the multiregion integrated classification proposed by Sachnev et al. [17] and Craddock et al. [18].

This paper focuses on the diagnosis and lesion localization of MRI data for ADHD-200. The MRI data contained 938 samples provided by different medical institutions, each of which could be converted to a three-dimensional matrix of 121×145×121. In this paper, on the basis of the assumption of local volume changes in the putamen and globus pallidus for a patient sample, diagnosis and lesion region detection were complemented using a random forests algorithm. The random forests classifier was trained on the segmented two-dimension slices of the matrix along the same position in each of the three directions. Then the diagnosis was determined on the testing data on the basis of the following: (1) The slices are labeled as a disorder if *k* adjacent slices were decided to be a disorder; and (2) the intersection of slices from three directions was determined to be the position of the disorder. The paper’s contributions are:

(1) On the basis of the 3D structure of MRI data, a multi-dimensional slice segmental method was proposed to enlarge the number of sample size and deduce the dimension. By integrating the position information of each slice, the spatial information of the data can be obtained to identify lesion regions.
(2) Slices were labeled to be a disorder if *k* adjacent slices were decided to be a disorder to improve the final accuracy of classification. The optimal *k* is 3.
(3) Classification accuracy was improved up to 75.4% in the full datasets, and it also reached a high point (of 81.82%) within the existing methods on a single sub-data set for the Kennedy Krieger Institute (KKI). Two suspected disorder regions were also detected, one of which perfectly matched the region found by Li et al. [16], and another suspected region was found at the first time.

## 2. Material

The ADHD-200 data used in this paper were ADHD MRI data. The dataset is available from http://fcon_1000.projects.nitrc.org/indi/adhd200/ accessed on 1 July 2011. The dataset included normal controls (Typically Developing, TD) and ADHD patients. ADHD-200 is composed of many sub-datasets provided by multiple research institution sites. The dataset consisted of 938 samples—579 normal controls and 359 ADHD patients—in which the patients’ data consisted of 262 samples and 94 samples from NYU and KKI, respectively.

Each MRI sample could be transferred as a 3D gray value tensor *X* of size 121×145×121, that is, we could treat each sample as a cube with length, width and height of 121, 145 and 121 respectively, i.e., the cross-section, sagittal plane and coronal plane of *X* were segmented from a medical perspective. *y* is sample labels indicating the appearance of ADHD.

## 3. Methods

The procedure of the proposed method consisted of the segmentation of a 3D MRI sample, classification based on random forest and diagnosis decision based on interaction.

### 3.1. Segmentation of the 3D-MRI Sample

**Figure 1:**
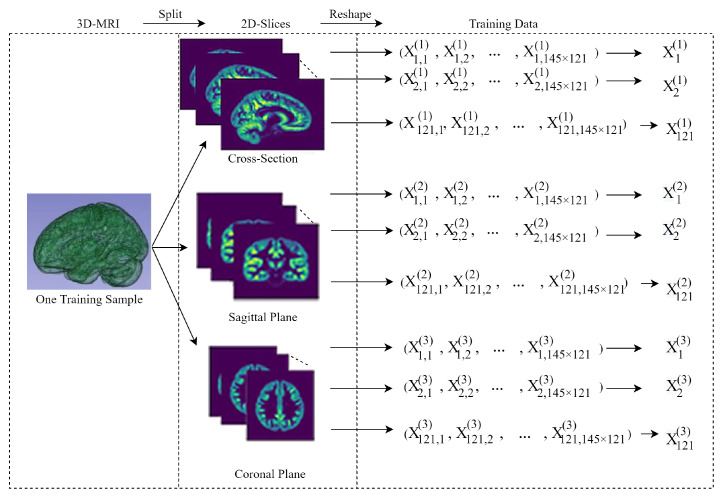
Segmentation of the 3D-magnetic resonance imaging (MRI) training sample.

We placed 3D-MRI samples in a standard space used to express neuroimaging data, and established a spatial coordinate system on the transverse section, the median sagittal section and the coronal section [19]. The 3D-MRI sample was continuously segmented in three directions; then we obtained a corresponding number of slices in each segmentation direction, and each slice corresponded to a 2D MRI slice. If the segmentation procedure proceeded continuously in any fixed direction, we would obtain continuous 2D images of different positions of the brain in the same object. For instance, in the first position of the cross-section, the sample was segmented into 938 2D images (of 145×121 size for each 2D image). Then each 2D image was reshaped into a vector. Generally, the *m*th position of one sample *X* in the first direction is reformed to a vector $X_{m}^{\left(1\right)}$ with one row and 145×121 columns, where m=1,2,…,121. The same procedure can be complemented in different positions of MRI data. The details of this procedure were illustrated in Figure 1.

The slice label must be assigned to a specific sample in supervised learning. In the real practise, the lesion region of the brain is a part or parts of the brain volume. Since it is not clear where exactly the disorder occurs, any region in the brain could be regarded as the disorder region, therefore every slice is labeled 1 if a diagnosis of ADHD is obtained in the training sample, even if the slice corresponds to a normal part of the patient. In fact, if the slices of disorder are well marked, the classifier can fully learn the features in the sample and eventually obtain the correct classification label. Otherwise, if the slices in the area without disorder are labeled incorrectly, it can be considered as noise in the following classification procedure and verified through the threshold selection procedure.

### 3.2. Classification of 2D Slices Based on Random Forests

To evaluation the performance of the proposed method, the 938 data were partitioned into 10 folds of sub-samples, and each sub-sample was assigned to the training (with size $n_{Tr}$) or testing datasets (with size $n_{Te}$) randomly by using 10-fold cross-validation.

Random forests builds a large collection of decorrelated trees and then averages them. Here the random forests model was used to recognize the classification criterion concerning use of the training set, and then to obtain labels for all the slices in each of the three directions of the 3D matrix of the MRI in the testing set. The 2D images from the same positions and the same directions in the training set were composed into a designed input matrix of the random forest model, and then the well-trained model obtained by training 2D images was imputed to identify the 2D images of the corresponding position of the same direction in the testing set, and lastly, the position information of each slice in three directions and the corresponding predicted label were reconstructed into a decision matrix $Y_{i}\left(i=1,2,3\right)$. So for each test sample, three decision matrices in three directions were obtained, each component of the matrix corresponded to the classification result of the test slice at the specific dimension.

When the classification accuracy of the testing set in a certain dimension reached higher, the model could perform better in the whole classification task. The resulting classification performance was very robust and more accurate by setting n_estimators = 700, max_depth = 100, min_samples_split = 2 (referred to as the RandomForestClassifier function in Python 3.6 Sklearn library), where n_estimators, max_depth and min_samples_split represents the number of trees, the maximum depth of each tree and the minimum number of branches, respectively.

**Figure 2:**
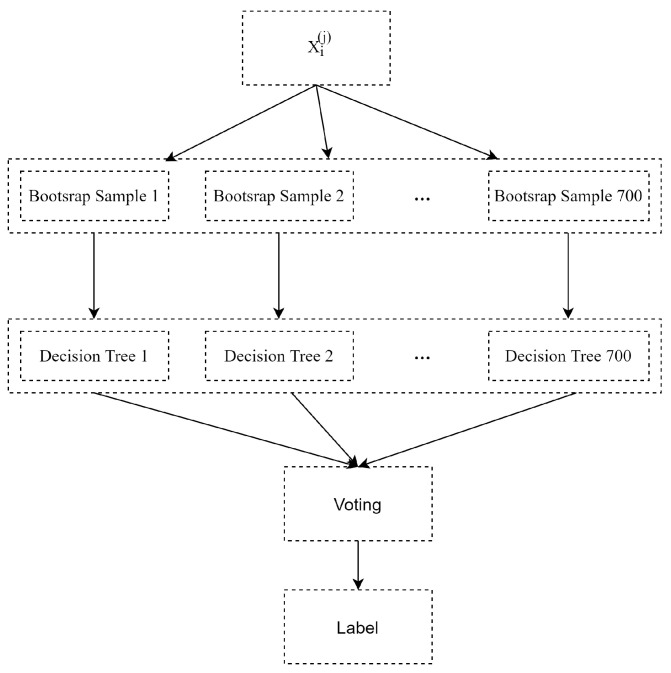
The flow chart of the random forest classifier.

The procedure of random forests classification on the *i*-th position of the *j*-th direction is illustrated in Figure 2. In Figure 2, $X_{i}^{\left(j\right)}$ is a vector of the *i*-th position of the training sample *X* in the *j*-th direction, as shown in Figure 1. The procedure has three steps:

**Step** **1:** Randomly select sub-samples from training samples by sampling with replacement as a bootstrap sample for 700 iterations.
**Step** **2:** Train 700 decision trees by using different bootstrap samples.
**Step** **3:** Make a classification for testing samples by using well-trained decision trees and exploit a majority vote on the classification results.

### 3.3. Diagnosis and Localization on Testing Sample

**Figure 3:**
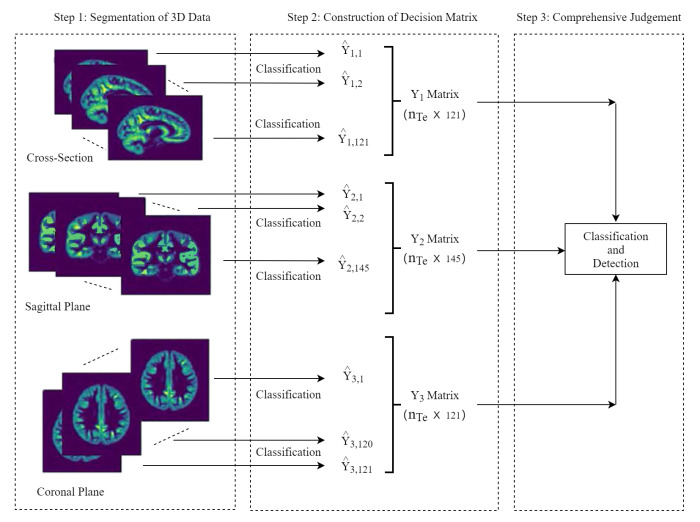
Flow chart of the proposed method on the testing sample.

Based on the decision matrix $Y_{i}$ formed by each testing set sample obtained by the random forest model, a final identification of disorder (classification) and detection of lesion region was implemented. For each sample from the testing set, upon the segmentation aforementioned, a number of 121, 145 and 121 slices could be obtained in each of three directions, respectively. Therefore, a prediction matrix sized 121×145×121 could be restructured from the decision information. Diagnosis and location of the suspected disorder region were determined by the intersection of lesion slices from three directions. The procedure of the proposed method on the testing sample is illustrated in Figure 3.

Since the disorder is a part volume of brain MRI, the disorder information can be revealed in a local 3D region, so slices from a testing sample can be labeled as 0 (normal) or 1 (disorder). In this sense, if there are multiple consecutive slices that are judged to be a disorder in a certain dimension, the area composed of these consecutive slices is a suspected lesion area. At the same time, if there are such suspected areas in three dimensions that then intersect in the three-dimensional orthogonal space, the final three-dimensional lesion area is determined. The location of this area in the brain can be determined according to the specific numbers of these consecutive slices in each dimension. The number of consecutive slices k(k>1) is an unknown threshold to be specified.

## 4. Results

This section presents the performance of the segmentation-reshape-RF method in ADHD-200. The grid search method was used to determine the model parameters. As described in Section 3.2, all parameters ensured that the random forest model could achieve the highest classification accuracy in the classification process of a single slice. In addition, the selection of the threshold *k* stemmed out of the same purpose, that is, the highest classification accuracy needed to be achieved in the process of sample classification.

### 4.1. Selection of Threshold k

The measurement of classification accuracy (ACC) was chosen to give an appropriate value of *k*. Table 1 shows the results with respect to the parameter *k*. Here the threshold *k* was selected to give the highest classification accuracy. It could be seen from Table 1 that when *k* was 3, the accuracy of classification reached a maximum value. So k=3 was the appropriate selection.

### 4.2. Diagnosis Based on Classification

The performance of the proposed method was compared with the algorithm that performed well in the literature. For comparison, we chose research methods that used the same sample data and sample size as the one we used. In the presentation of classification results, we compared two different methods.

(1) The first two methods take into account the heterogeneity between the datasets, and the search process is based on the sub-datasets NY and KKI.
  Social Network [9] utilized social network to extract the features of the ADHD-200 resting state function MRI (fMRI) data, and the classification is implemented by support vector machine classifier.
  Multi-Level [13] exploited a decision-trees-based multi-level approach, where the decision tree was constructed upon a subset of significant features.
(2) The second type of research method is entirely based on the whole ADHD-200 dataset.
  FV + Demo [20] developed a support vector machine model to classify ADHD patients from typically developing controls (TDCs) using the regional brain volumes as predictors. The classifier utilized the highlighted 10 brain regions most distinguished in discriminating between ADHD patients and TDCs.
  In Reg-Tucker [16], the original data was decomposed by the tucker method first, and then the decomposed features were classified and reconstructed by the tensor regression method.
  Tensor LogitBoost [21] used the same feature extraction method as [16], it alternatively used Logit Boost algorithm in the classification procedure, which achieved better classification results compared with Li’s idea [16].
  3D-CNN [7] extracted a meaningful 3D low-level feature from function MRI (fMRI) and structural MRI (sMRI) data, and then used a multi-modality CNN architecture to combine fMRI and sMRI features. Since this method kept the low-level features as three-order tensors, the spatial information of the data was well preserved.

Table 2a,b show the comparisons of the proposed methods with FV + Demo [20], Reg-Tucker [16], Tensor LogitBoost [21] and 3D-CNN [7]. Their classification accuracy on full data was 68.6%, 68%, 69% and 69.15%, respectively. Our proposed method had a best classification accuracy of 75.44% on full data. Moreover, we also achieved the best results on a single dataset provided by NYU and KKI, with accuracies of 70.73% and 81.82%, respectively.

**Figure 4:**
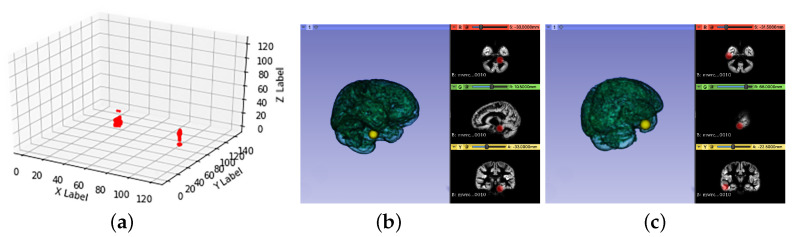
Visualization of the comment disorder region of nine patients. (**a**) in three-dimensional coordinate space; (**b**,**c**) two different suspected lesions in 3D and 2D images.

**Figure 5:**
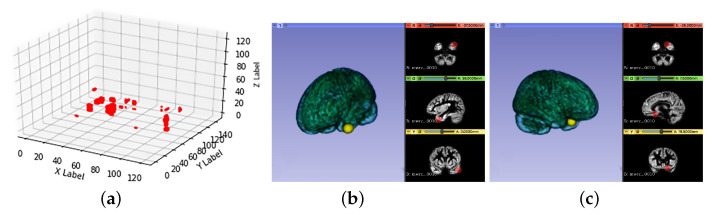
Visualization of the comment disorder region of eight patients. (**a**) in three-dimensional coordinate space; (**b**,**c**) two different suspected lesions in 3D and 2D images.

Due to the differences in individuals’ brains, lesion location was also different. Here the intersection of different individual lesion regions was considered to be the common incidence region of ADHD. In order to show the common disorder area more clearly, the visualization images of the intersection of the lesion regions of 9 and 8 individuals were presented in Figure 4 and Figure 5. Figure 4a and Figure 5a show the results of common pathogenic regions obtained from our proposed segmentation-reshape-RF method for nine patients and eight patients in the three-dimensional coordinate space. Figure 4b,c and Figure 5b,c are 3D visualizations of these common pathogenic regions made using 3D Slicer software (https://www.slicer.org, accessed on 30 September 2020); the left half shows the brain’s 3D model and the responding position of the disorder region (yellow ball) in the three-dimensional perspective, and the right half the specific position of the disorder region (in red) from three different viewpoints.

As shown in Figure 4a and Figure 5a, it is clear that the eight patients had more common pathogenic regions than the nine patients did, because these regions were acquired from the intersection of some patients’ disorder areas. More surprisingly, Figure 5b,c show two additional regions on top of those in Figure 4. The disorder regions found in Figure 5c perfectly matched the suspected lesion region found by Li et al. [16], where the author used the Tucker regression method to find the potential lesion region. This finding proved the effectiveness of our method and consistency with the referenced results.

## 5. Discussion

This neuro-developmental disorder impairs the growth of children. Therefore, early detection and location of lesions is beneficial to the healthy development of a child’s body and mind. We proposed a random-forest-based classification technique that depended on MRI segmentation and orthogonal interaction methods. The performance was promising on the dataset from New York University (NYU), Kennedy Krieger Institute (KKI) and full ADHD-200 datasets. Our research is based on the assumption of local volume changes in the putamen and globus pallidus; however, some studies also suggested a global reduction in WM volume associated with ADHD, or in addition to the potential microstructural and functional changes that would not be detectable from structural MRI alone. This assumption was a possible limitation in this research.

## 6. Conclusions

This paper was mainly devoted to the disorder classification and suspected disorder-region localization of MRI samples from ADHD. A multi-dimensional segmentation method was carried to decompose the 3D tensor sample as a collection of slices. Considering the spatial structure of the MRI, random forest classifiers were separately trained in the different positions of different orthogonal directions, and then applied to the same position as the testing set. Compared with other dimensionality reduction methods, our method effectively retained the original spatial information of the data, reduced the dimension and enlarged the sample size, so it had a reliable effect on disorder region detection. The assumption of a lesion decision based on *k* adjacent slices can effectively reduce the misclassification. The performance on ADHD illustrated the superiority of the proposed method; this approach could be very useful from a medical point of view because such models can be applied to any other neurophysiological disease or disorder. Regarding the high rates of comorbidities and difficult differential diagnoses, especially for ADHD, a reliable computer-aided diagnostic tool in a medical image will support clinical decisions.

## Figures and Tables

**Table 1:** Comparison of ACC under different grid.

*k*	2	3	4	5
ACC	0.743	**0.754**	0.695	0.678

“ACC”: Classification accuracy; “*k*”: Number of consecutive slices for the judgement of a disorder region. Bold number means the optimal one.

**Table 2:** The Comparison of Classification Accuracy (a) on sub-datasets NY and KKI and (b) on whole ADHD-200 dataset.

(a)		
**Method**	**NYU(41)**	**KKI(11)**
Social Network [9]	63.75%	78.21%
Multi-Level [13]	58%	-
3D-CNN [7]	70.5%	72.82%
Proposed Method	**70.73%**	**81.82%**
(**b**)		
**Method**	**ADHD-200(171)**	
FV + Demo [20]	68.6%	
Reg-Tucker [16]	68%	
Tensor LogitBoost [21]	69%	
3D-CNN [7]	69.15%	
Proposed Method	**75.44%**	

“-”: There is no results in corresponding report; “NYU”: data from New York University; “KKI”: data from Kennedy Krieger Institute; Bold numbers represent the optimal ones.

## Data Availability

The dataset is available publicly from http://fcon_1000.projects.nitrc.org/indi/adhd200/ accessed on 1 July 2011.
